# Nanoparticle platform preferentially targeting liver sinusoidal endothelial cells induces tolerance in CD4+ T cell-mediated disease models

**DOI:** 10.3389/fimmu.2025.1542380

**Published:** 2025-03-17

**Authors:** Shu-Hung Wang, Isabelle Serr, Reinaldo Digigow, Barbara Metzler, Alexey Surnov, Cornelia Gottwick, Muhammad Alsamman, Daria Krzikalla, Markus Heine, Miriam Zahlten, Agata Widera, Disha Mungalpara, Muharrem Şeleci, Marco Fanzutti, Lígia Margarida Marques Mesquita, Anna-Lisa Vocaturo, Johannes Herkel, Antonella Carambia, Christian Schröter, Dikran Sarko, Johannes Pohlner, Carolin Daniel, Cristina de Min, Sabine Fleischer

**Affiliations:** ^1^ Department of Clinical Development, Topas Therapeutics GmbH, Hamburg, Germany; ^2^ Research Unit Type 1 Diabetes Immunology, Helmholtz Diabetes Center at Helmholtz Zentrum München, German Research Center for Environmental Health, Neuherberg, Germany; ^3^ German Center for Diabetes Research (DZD), Neuherberg, Germany; ^4^ Department of Chemistry, Manufacturing & Controls, Topas Therapeutics GmbH, Hamburg, Germany; ^5^ Department of Preclinical Development, Topas Therapeutics GmbH, Hamburg, Germany; ^6^ Department of Medicine, University Medical Centre Hamburg-Eppendorf, Hamburg, Germany; ^7^ Department of Biochemistry and Molecular Cell Biology (N30), University Medical Centre Hamburg-Eppendorf, Hamburg, Germany; ^8^ Hamburg Centre for Translational Immunology (HCTI), University Medical Centre Hamburg-Eppendorf, Hamburg, Germany; ^9^ Division of Clinical Pharmacology, Department of Medicine IV, Ludwig Maximilian University of Munich, Munich, Germany

**Keywords:** tolerance, nanoparticles, liver sinusoidal endothelial cells, antigen-specific immunotherapy, regulatory T cells, autoimmune diseases, T cell anergy

## Abstract

**Introduction:**

Treating autoimmune diseases without nonspecific immunosuppression remains challenging. To prevent or treat these conditions through targeted immunotherapy, we developed a clinical-stage nanoparticle platform that leverages the tolerogenic capacity of liver sinusoidal endothelial cells (LSECs) to restore antigen-specific immune tolerance.

**Methods:**

*In vivo* efficacy was evaluated in various CD4^+^ T cell-mediated disease models, including preventive and therapeutic models of myelin oligodendrocyte glycoprotein-induced experimental autoimmune encephalomyelitis (EAE), ovalbumin-sensitized delayed-type hypersensitivity (DTH), and the spontaneous type 1 diabetes model. Nanoparticle-induced antigen-specific immune responses were also analyzed through adoptive transfers of 2D2 transgenic T cells into wild-type mice, followed by nanoparticle administration.

**Results:**

The peptide-conjugated nanoparticles displayed a uniform size distribution (25–30 nm). Their coupling efficiency for peptides with unfavorable physicochemical properties was significantly enhanced by a proprietary linker technology. Preferential LSEC targeting of nanoparticles coupled with fluorescently labeled peptides was confirmed via intravital microscopy and flow cytometry. Intravenous nanoparticle administration significantly reduced disease severity and demyelination in EAE, independent of prednisone at maintenance doses, and suppressed target tissue inflammation in the DTH model. Furthermore, prophylactic administration of a mixture of nanoparticles coupled with five autoantigenic peptides significantly lowered the hyperglycemia incidence of the non-obese diabetic mice. Mechanistically, the tolerizing effects were associated with the induction of antigen-specific regulatory T cells and T cell anergy, which counteract proinflammatory T cells in the target tissue.

**Conclusion:**

Our findings demonstrate that peptide-loaded nanoparticles preferentially deliver disease-relevant peptides to LSECs, thereby inducing antigen-specific immune tolerance. This versatile clinical-stage nanoparticle platform holds promise for clinical application across multiple autoimmune diseases.

## Introduction

Autoimmune diseases (ADs), such as multiple sclerosis (MS) and type 1 diabetes (T1D), involve aberrant immune responses to self-antigens, resulting in chronic inflammation and tissue damage (1). The healthy immune system prevents autoimmunity by balancing self-tolerance and responses against foreign antigenic threats. Importantly, autoreactive T cells that escape thymic elimination require peripheral control mechanisms to render them harmless (2). The mechanisms for tolerizing autoreactive T cells comprise clonal deletion, anergy, or immunological ignorance (3). Moreover, peripheral tolerance of T cells is greatly influenced by regulatory T cells (Tregs), the maturation status or tolerogenic function of antigen-presenting cells (APCs), as well as immunoregulatory receptors such as cytotoxic T-lymphocyte antigen-4 (CTLA-4) and programmed death-1 (PD-1) (3). Failure of these control mechanisms can cause chronic autoimmunity.

While current therapies primarily depend on systemic immunosuppression, often accompanied by severe side effects, the ideal approach for treating ADs aims to restore immune tolerance towards the respective self-antigens the immune system mistakenly targets (4). Therefore, boosting internal tolerogenic pathways and inducing self-tolerance are primary objectives in developing curative AD therapies (5).

Recently, various nanomedicine strategies for treating ADs have been proposed (6). Among those having reached clinical stages, one prominent strategy employs nanoparticles carrying pharmacological agents capable of inducing tolerogenic APCs, tackling anti-drug antibody responses in patients (7). Other approaches involve antigen delivery to leverage liver tolerance mechanisms, utilizing nanoparticles mimicking apoptotic cells or antigens bearing glycosylation signatures (8, 9). These latter strategies have been tested in coeliac disease patients, showing initial signs of effectiveness (10, 11). Here, we present an optimized clinical-stage nanoparticle-based approach that specifically targets liver sinusoidal endothelial cells (LSECs).

The liver is widely recognized for its remarkable ability to tolerize foreign antigens, a necessity considering its constant exposure to antigens from the diet or gut microbiome (12). While various non-parenchymal liver cells (NPLCs) exhibit tolerogenic capacities, LSECs outnumber Kupffer cells by approximately 2.5 times and excel in promoting tolerance even under inflammatory conditions (12).

Delivering disease-relevant peptides to LSECs by an early version of our nanoparticles significantly suppressed autoimmune pathology in relevant animal models (13). For clinical use, we subsequently optimized our particles with a new coating polymer to improve biodegradability and manufacturability. This updated version showed potent tolerizing effects on antigen-specific CD8^+^ T cells in mice (14). To warrant a broad range of clinical applications, we further refined the formulation and peptide carrier functions of these nanoparticles, now called “Topas Particle Conjugates” (TPCs). Here, we present the results of this optimization process using a proprietary linker technology for diverse peptide conjugation, confirming that the updated TPCs preferentially target LSECs and effectively induce peptide-specific T cell tolerance in clinically relevant CD4^+^ T cell-mediated disease models. Due to their unique physicochemical properties, TPCs provide a versatile platform for delivering diverse antigenic peptides to highly tolerogenic LSECs, leveraging the precision of nanomedicine to induce antigen-specific tolerance in ADs.

## Results

### Nanosized antigen carriers developed for tolerance induction

**Figure 1:**
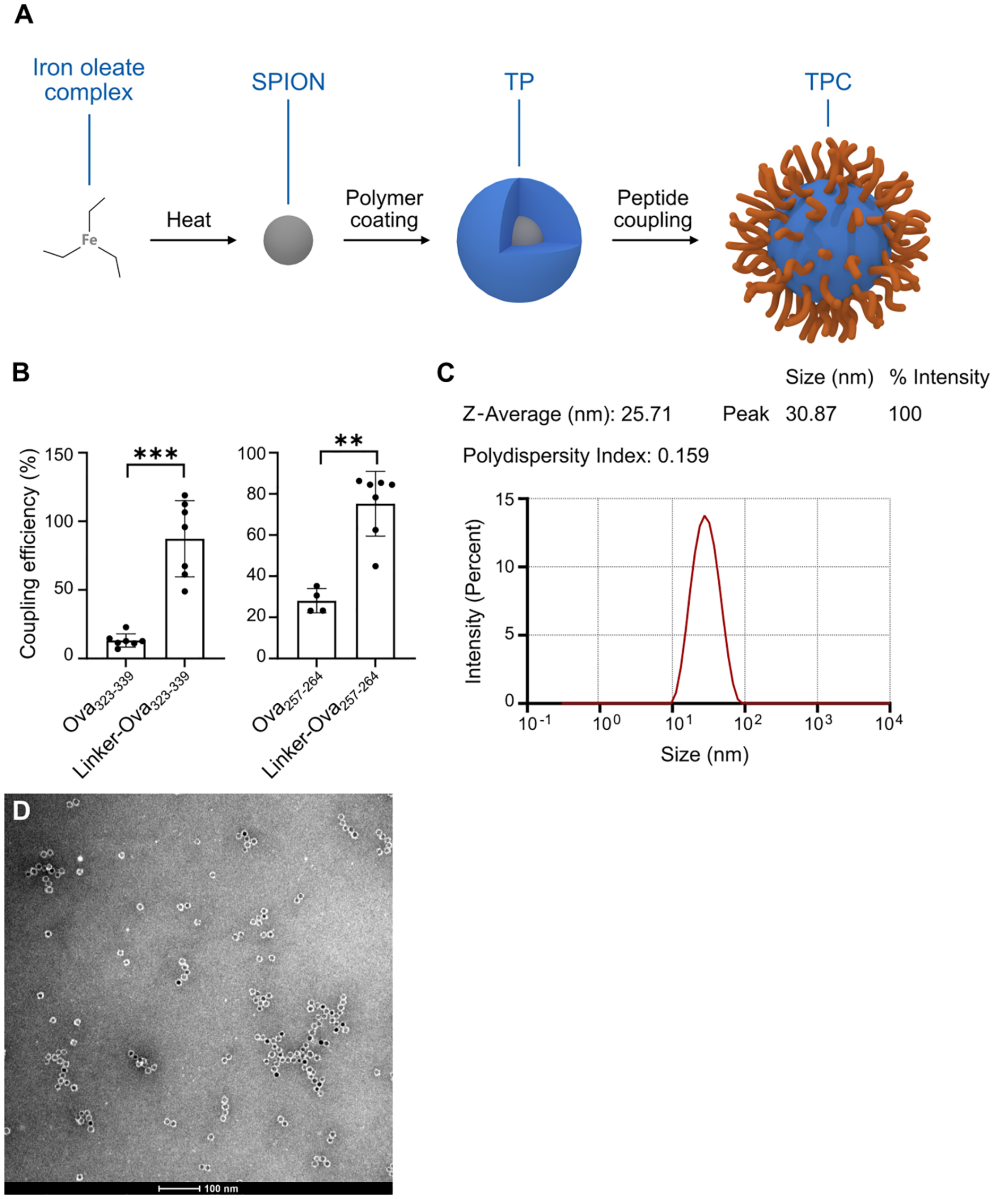
TPCs display uniform size distribution. **(A)** Production process scheme of TPCs. **(B)** Coupling efficiency of Ova peptides with and without the linker. **(C)** Dynamic light scattering: Size distribution of TPCs, average size (nm), and polydispersity index. **(D)** Transmission electron microscopy image of TPCs. Graphs show means ± SD. Data are representative of 7–8 experiments. **p < 0.01; ***p < 0.001 by Mann-Whitney test for the differences in coupling efficiency.

Building on former preclinical versions of the nanosized antigen carriers (13, 14), we have effectively advanced this peptide delivery system to LSECs into a platform technology for clinical application. Superparamagnetic iron oxide nanoparticles (SPIONs) are coated with low-molecular-weight poly-maleic-acid-*alt*-octadecene polymer (**Figure 1A**). The polymer-coated SPIONs, termed “Topas Particles” (TPs), serve as a scaffold for conjugating antigenic peptides to their surface to generate TPCs. Importantly, the small size of the TPs and their high surface density of carboxylic groups, indicated by the negative ζ-potential, allow for high peptide loading, with >100 peptide molecules per TPC. Regardless of the coupled peptides, TPCs consistently show an overall negative ζ-potential (≤ –30 mV).

The peptide conjugation process utilizes EDC (1-ethyl-3-(3-dimethylaminopropyl)-carbodiimide) chemistry, linking the N-terminal amine of the peptide to the available carboxylic groups on the TP surface. To maximize flexibility in selecting a broad variety of peptides for clinical application, we developed a proprietary linker technology specifically for peptides with unfavorable conjugation characteristics that preclude EDC coupling alone. Attaching a linker to the N-terminus of the peptide during synthesis adjusts its net charge, isoelectric point, and water solubility, thereby enabling efficient EDC coupling. For instance, the linker converts the negative charge of ovalbumin (Ova)_323-339_ peptides to positive, facilitating their coupling with negatively charged TPs, while also increasing the hydrophilicity of Ova_257-264_ (**Table 1**). This resulted in coupling efficiency improvements of approximately 75% for Ova_323-339_ and nearly 50% for Ova_257-264_ (**Figure 1B**). We subsequently employed in-silico peptide screening to predict which peptides required the linker for efficient coupling, minimizing unnecessary in-lab testing and streamlining development.

**Table 1:** Physicochemical properties of peptides with and without a linker.

Peptide	Net charge at pH 9	Isoelectric point	GRAVY*
ISQAVHAAHAEINEAGR (Ova_323-339_)	-1.17	6.00	-0.23
Linker-ISQAVHAAHAEINEAGR	0.83	9.52	-0.68
SIINFEKL (Ova_257-264_)	-0.20	5.72	0.49
Linker-SIINFEKL	1.76	10.84	-0.51

The physicochemical properties of peptides were calculated using an online tool (https://www.biosynth.com/peptide-calculator).

*GRAVY: Grand Average of Hydropathy. The score is determined by summing the hydropathy values of all the amino acids and then dividing that total by the number of residues. Peptides with negative GRAVY values are hydrophilic.

Each TPC carries around 100 copies of one specific peptide. To apply multiple peptides simultaneously, individual TPCs are mixed to deliver “Topas Particle Mixtures” (TPMs). Formulated in Mannitol/Tris/Lactate buffer, TPCs and TPMs are compatible with intravenous administration to patients. Moreover, TPC manufacturing has been successfully scaled up to meet the GMP-compliant production requirements for human use.

Measures were implemented to ensure minimal size variation and a low polydispersity index of TPCs. Specifically, analysis using transmission electron microscopy and dynamic light scattering revealed that both TPs and TPCs exhibited a mean diameter (Z-average) of 25–30 nm, with a uniform size distribution indicated by low polydispersity index values (**Figures 1C, D**), independent of the peptide load. Of note, TPCs did not form aggregates when exposed to serum or plasma *in vitro*.

### Preferential LSEC targeting

**Figure 2:**
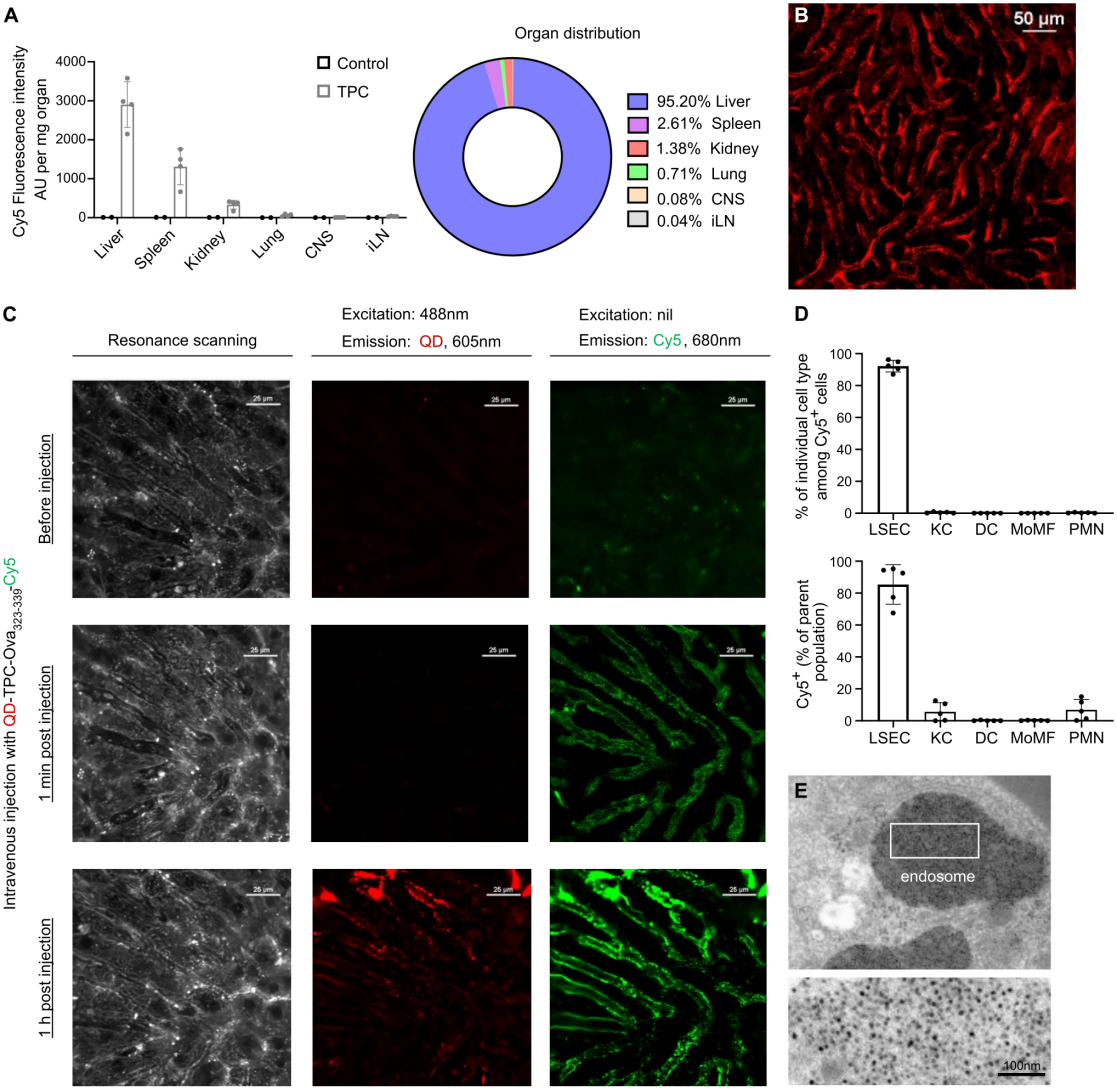
TPCs primarily accumulate in the liver, predominantly within LSECs. **(A)** Organ distribution of TPCs. Ten minutes after the intravenous administration of TPC-gliadin-Cy5, the fluorescence intensity in different organ lysates was analyzed. **(B)** Intravital microscopy of the liver. **(C)** The liver of C57BL/6 mice was examined under intravital microscopy before and after i.v. injection with QD-TPC-Ova_323-339_-Cy5. Excitation of QD at 488 nm with Cy5 emission at 680 nm indicated the presence of FRET. Baseline (*upper*), 1 min post-injection (*middle*), and 1 h post-injection (*lower*). **(D)** Ten minutes after the intravenous administration of TPC-gliadin-Cy5, flow cytometry was undertaken to analyze two parameters (gating strategy: **Supplementary Figure S2A**): the percentage of each NPLC type among Cy5^+^ cells (*upper* panel) and the percentage of Cy5^+^ cells within each NPLC type (*lower* panel). **(E)** Detection of TPC-like particles (i.e., former version of TPCs) within LSEC endosomes by electron microscopy of minipig liver. CNS: central nervous system; iLN: inguinal lymph nodes; KC: Kupffer cells; DC: dendritic cells; MoMF: monocyte-derived macrophages; PMN: polymorphonuclear neutrophils. Graphs show means ± SD of five mice. A representative experiment of two studies that resulted in similar outcomes is shown for **(A, B, D)**. FRET analysis **(C)** and electron microscopy **(E)** were done once.

To investigate organ distribution, we intravenously injected TPCs coupled with Cyanine-5 (Cy5)-labelled gliadin peptides (TPC-gliadin-Cy5) into wild-type mice. Among the organs assessed, the liver displayed by far the highest fluorescence intensity, whereas the spleen and kidneys showed substantially lower levels (**Figure 2A**). Using intravital microscopy, fluorescence signals were specifically detected along the liver sinusoidal lining, corresponding to the anatomical location of LSECs, with no fluorescence signals in hepatocytes or stellate cells (**Figure 2B**). Moreover, to assess intracellular peptide dissociation from TPCs within the liver up to one hour after administration, we generated nanoparticles incorporating quantum dots instead of iron core (QD-TP), Ova_323-339_-Cy5, TP coupled with Ova_323-339_-Cy5 (TPC-Ova_323-339_-Cy5), and QD-TPC-Ova_323-339_-Cy5. Following injections with these nanoparticles, wild-type mice underwent intravital microscopy at different timepoints (**Figure 2C**). Förster resonance energy transfer (FRET) indicated the proximity between the excited donor fluorophore QD and its acceptor Cy5, serving as a proxy for intact QD-TPC-Ova_323-339_-Cy5 (**Supplementary Figure S1A**). We employed FRET to demonstrate the uptake of intact TPCs by the liver and their subsequent peptide release. While fluorescence along the liver sinusoidal lining initially originated solely from Cy5 due to FRET, QD emission became detectable 1 h post-injection. Conversely, FRET was absent upon injection of TPC-Ova_323-339_-Cy5 (lacking energy donors) or QD-TP plus uncoupled Ova_323-339_-Cy5 (lacking donor-acceptor proximity) (**Supplementary Figures S1, B, C**). Our data confirm that TPCs are rapidly taken up as intact particle-peptide conjugates, followed by gradual peptide release from TPCs as evidenced one hour after injection. This predominantly occurs in LSECs, where the most prominent TPC-uptake is visualized by their characteristic anatomical location.

Indeed, among NPLCs, LSECs exhibited a preferential uptake of TPCs (92.2% ± 1.64%), with >80% of LSECs showing uptake (**Figure 2D**). This cellular targeting preference was further validated by electron microscopy of the minipig liver, revealing the presence of TPCs within endosomes of LSECs thanks to the contrast-enhancing properties of the TPC iron-oxide core (**Figure 2E**). As endosomes represent the subcellular compartment where antigen processing and peptide loading onto MHC-II (major histocompatibility complex class-II) molecules take place (15), this observation corroborates that TPC-delivered peptides can be readily processed by LSECs similarly to blood-borne antigens.

**Figure 3:**
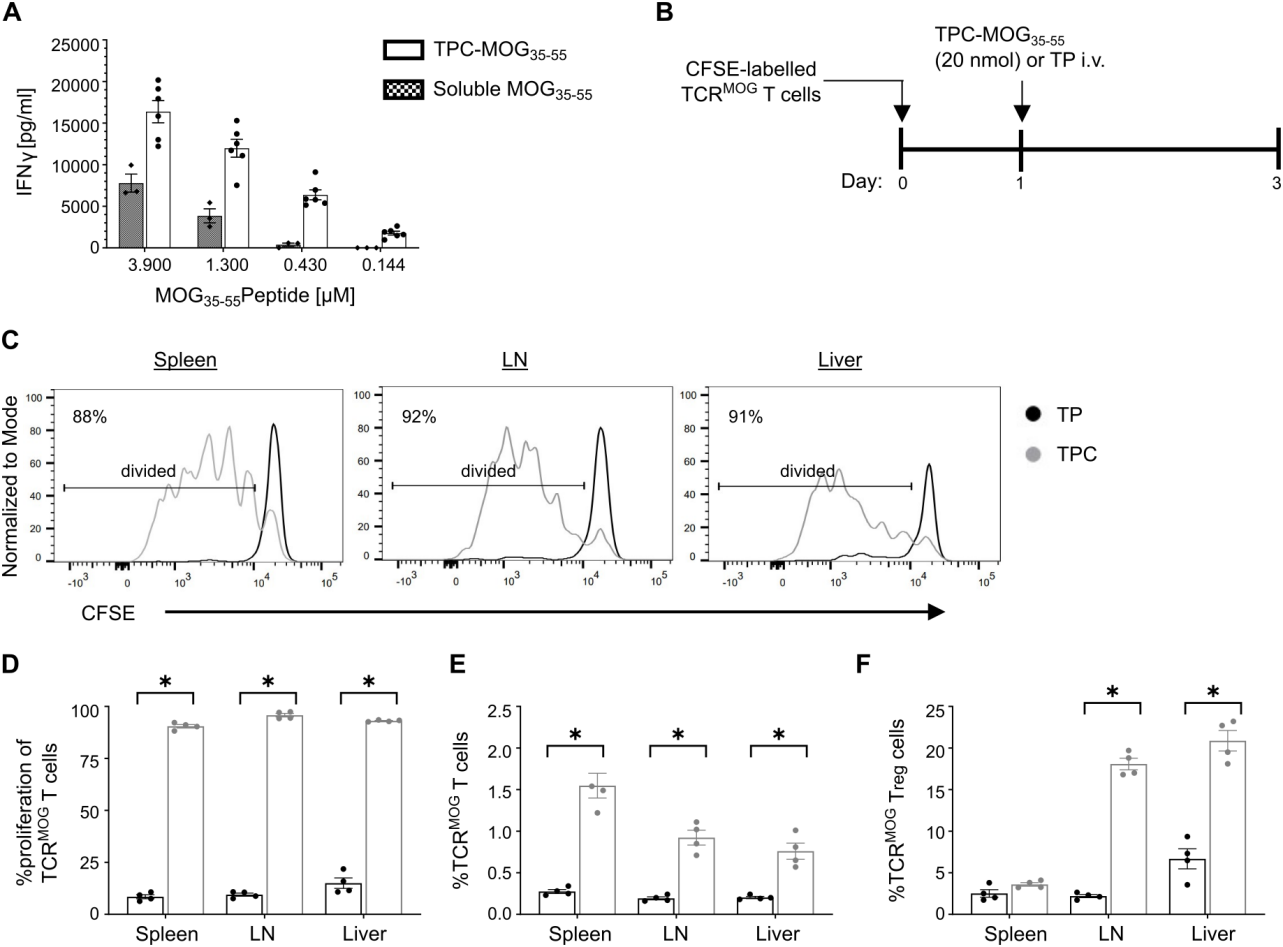
Antigen presentation of TPCs by APCs results in antigen-specific T cell activation and Treg induction. **(A)** Lymphocytes were isolated from spleen and lymph nodes of 2D2 mice and stimulated *in vitro* for 72 h with TPC-MOG_35–55_ or soluble MOG_35–55_ peptides in various concentrations. The level of IFNγ in the supernatant was measured by ELISA. **(B–F)** TCR^MOG^ T cells were labelled with carboxyfluorescein diacetate succinimidyl ester (CFSE) and adoptively transferred into wild-type mice, followed by treatment with TP or TPC-MOG_35-55_ (20 nmol) (N = 4 per group). Organs were harvested two days after treatment and analyzed using flow cytometry. Study design **(B)**. Representative CFSE intensity histograms of TCR^MOG^ T cells recovered from different organs of animals treated with either TP (*black*) or TPC (*grey*) **(C)**. Proliferation (i.e., CFSE dilution) **(D)** and frequency **(E)** of TCR^MOG^ T cells in different organs. Frequency of TCR^MOG^ Treg cells in different organs **(F).** Graphs show means ± SD. *p < 0.05 by Mann-Whitney test.

As antigen-specific T cell activation is regarded as a first step for tolerance induction, we aimed to validate the biological functionality of TPCs in promoting a peptide-specific CD4^+^ T cell response upon antigen presentation. To this end, we performed an *in vitro* stimulation assay using T cell receptor (TCR)-transgenic splenocytes specific to myelin oligodendrocyte glycoprotein (MOG)_35-55_ peptide. Incubating these cells with TPC-MOG_35-55_ resulted in dose-dependent IFN-γ secretion (**Figure 3A**). Similarly, TPC-MOG_35-55_ also induced approximately 80% greater proliferation of MOG-specific TCR-transgenic CD4^+^ (TCR^MOG^) T cells *in vivo* compared to TP treatment, resulting in a significantly higher frequency of TCR^MOG^ T cells (**Figures 3B–E**). MOG-specific T cell activation was observed in both the lymphoid organs and liver, while an increased frequency of TCR^MOG^ Tregs (CD3^+^CD4^+^Foxp3^+^) was noted in the liver but not the spleen, suggesting that tolerance initiation occurs in the liver (**Figure 3F**).

### Tolerance induction across different animal models

**Figure 4:**
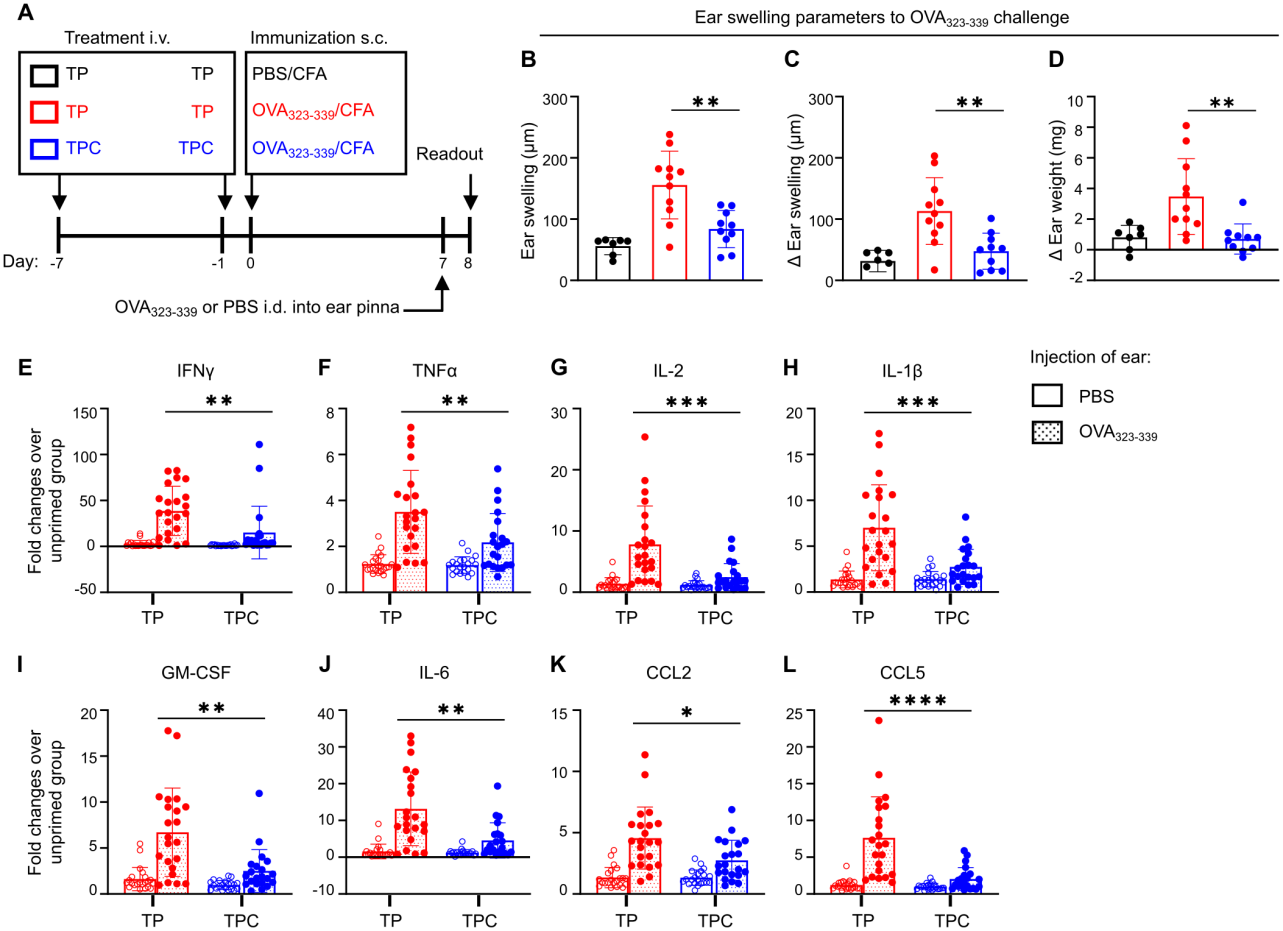
TPCs alleviate DTH responses and reduce the overall expression of proinflammatory cytokines and chemokines. BALB/C mice were injected i.v. with TP or TPC-OVA_323-339_ (14 nmol) 7d and 1d before immunization with OVA_323-339_ peptides plus complete Freund’s adjuvant (CFA) (n=10-11 per group). **(A)** Experimental setup. The negative control group (*black*) received TP i.v. on Day -7 and Day -1 and immunization s.c. with PBS plus CFA on Day 0. The non-tolerized group (*red*) received TP i.v. on Day -7 and Day -1 and immunization s.c. with OVA_323-339_ peptide plus CFA on Day 0. The tolerized group (*blue*) received TPC-OVA_323-339_ i.v. on Day -7 and Day -1 and immunization s.c. with OVA_323-339_ peptide plus CFA on Day 0. All animals were injected intradermally (i.d.) with OVA_323-339_ peptides into the ear pinna to elicit DTH response, while PBS was injected i.d. into the contralateral ear pinna of each animal as an internal control. **(B–D)** DTH responses were determined 24h after i.d. injections of ears with the priming peptides, using three different parameters: Ear swelling **(B)**; ear swelling in response to PBS in the contralateral ear of the animals subtracted from each measure of ear swelling (Δ Ear swelling; **(C)**); weight of a biopsy from the OVA_323-339_ peptide-challenged ear minus weight of the contralateral control with PBS (Δ Ear weight; **(D)**). The contralateral ear serves as internal control to minimize the interindividual variability. **(E–L)**, Relative mRNA expression of proinflammatory cytokines and chemokines of the ear pinnae from OVA_323-339_ peptide-primed versus unprimed animals, shown as fold changes: IFNγ **(E)**, TNFα **(F)**, IL-2 **(G)**, IL-1β **(H)**, GM-CSF **(I)**, IL-6 **(J)**, CCL2 **(K)**, and CCL5 **(L)**. Graphs show means ± SD. Data are a summary of two independent experiments. *p < 0.05; **p < 0.01; ***p < 0.001; ****p < 0.0001. Mann-Whitney test for the differences in DTH responses and relative gene expression between TP and TPC groups.

Having established LSECs as a preferential target for TPC delivery, we proceeded to assess their capability of tolerance induction across various CD4^+^ T cell-dependent disease models. In the delayed-type hypersensitivity (DTH) model, TPC-Ova_323-339_ rendered Ova-specific CD4^+^ T cells unresponsive to Ova_323–339_ peptide upon intradermal challenge, with approximate 50% reductions in ear swelling and Δ ear swelling and a nearly 75% reduction in Δ ear weight (**Figures 4A–D**). This significantly reduced mRNA expression of proinflammatory cytokines and chemokines in Ova-challenged ears of TPC-treated compared to TP-treated animals (**Figures 4E–L**).

**Figure 5:**
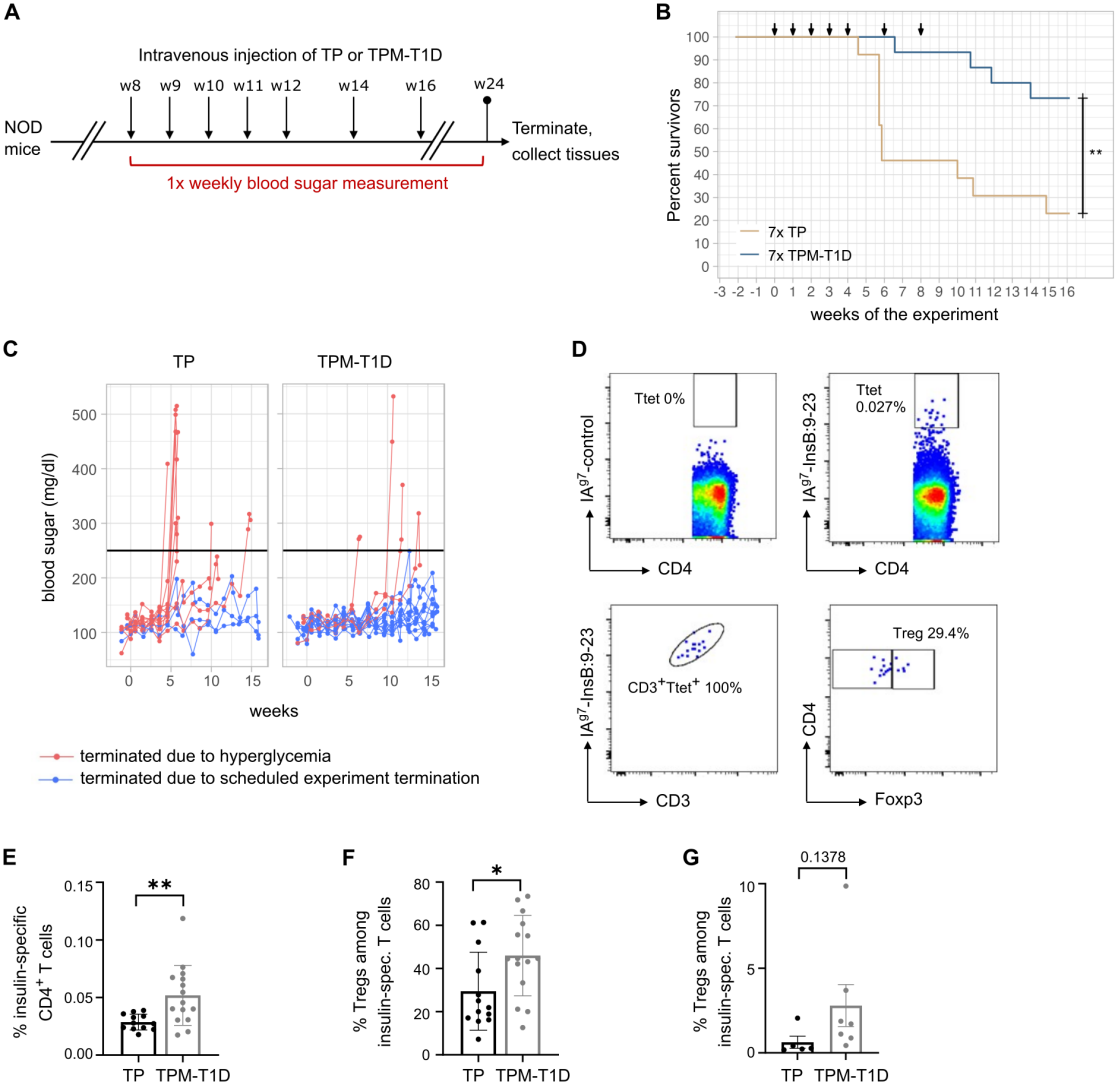
TPCs reduce onsets of hyperglycemia in NOD mice and increase insulin-specific Tregs. **(A)** Experimental setup. **(B)** Frequency of diabetes-free mice (“survivors”) per week of experiment. Arrows indicate timepoints of the injections. **(C)** Blood sugar levels throughout the experiment. The black line indicates the cutoff for hyperglycemia. **(D)** Representative plots of flow cytometry for IA^g7^-restricted control (*upper* panel, left) and InsB:9-23 tetramers (*upper* panel, right), correlation of tetramer staining with CD3 staining as quality control (*lower* panel, left) and gating on Foxp3^+^ Tregs among insulin-specific CD4^+^ T cells (*lower* panel, right). T_tet_: tetramer positive T cells. **(E)** Frequency of insulin-specific T cells among total CD4^+^ T cells in the spleen of mice treated with TP or TPM-T1D. **(F–G)** Frequency of Foxp3^+^ Tregs among insulin-specific T cells in the spleen **(F)** and pancreas **(G)** of mice treated with TP or TPM-T1D. Graphs show means ± SD of 13–15 **(A–F)** or 5-7 **(G)** mice per group. Data are a summary of two independent experiments **(A–F)** or one experiment **(G)**. *p < 0.05; **p < 0.01. Log-rank test for the survival analysis of diabetes-free animals; Mann-Whitney test for the differences in frequency of T cell subsets between animals treated with TP and TPM-T1D.

We further examined the efficacy of TPCs using a spontaneous mouse model of T1D: the non-obese diabetic (NOD) model. In NOD mice, the I-A^g7^ MHC-II molecule confers genetic susceptibility to T1D, with insulin-specific CD4^+^ T cells playing a crucial role in immunopathogenesis alongside other immune cells (16, 17). Considering the complexity of the autoantigenic responses, previous research suggests that a mixture of multiple autoantigenic peptides is required to induce tolerance in this model (18). Therefore, we selected five disease-relevant pancreatic antigenic peptides, one CD8^+^ and four CD4^+^ T cell epitopes, to create a mixture of TPCs called TPM-T1D (**Table 2**). Eight-week-old female NOD mice received seven injections of TP or TPM-T1D loaded with 50 nmol peptide in total (each TPC-peptide dosed with 10 nmol per injection) and were monitored until 24 weeks of age for diabetes incidence (**Figure 5A**). Multiple administrations of TPM-T1D significantly reduced the frequency of hyperglycemia onsets compared to TP treatment (**Figures 5B, C**). After treatment with TPM-T1D, insulin-specific CD4^+^ T cells in the spleen not only increased in numbers but notably entailed a roughly 20% higher proportion of Tregs compared to TP treatment (**Figures 5D–F**). In addition, we detected a clear trend of increased insulin-specific Tregs directly in the pancreas of treated animals (**Figure 5G**). These findings suggest that even when the disease involves multiple autoantigenic epitopes, a combination of selected peptides has the potential to induce tolerance in T1D. This treatment effect was associated with a significant accumulation of insulin-specific Tregs, possibly extending beyond antigen-specific suppression.

**Table 2:** Diabetogenic peptides used in TPM-T1D.

Peptide	Rationale	Sequence	TPC ID
Insulin beta chain (InsB) 9-23 (53)	Dominant pathogenic CD4 T cell epitope in both NOD and humans	SHLVEALYLVCGERG	TPC0013
Proinsulin p24-33 (54)	Early autoantigen in NOD mice	FFYTPMSRRE	TPC0115
IGRP206-214 (55)	Relevant CD8 autoantigen in NOD mice	VYLKTNVFL	TPC0041
2.5HIP (fused ChgA peptide) (56–58)	Agonistic for mouse and human islet-infiltrating T cells; neoantigen	DLQTLALWSRMDQLAK	TPC0038
6.9HIP (fused IAPP2) (56, 59)		DLQTLALNAARDPNR	TPC0039

ChgA, chromogranin A; HIP, hybrid insulin peptide; fusion of insulin to other disease-relevant peptides. IAPP2, islet amyloid polypeptide 2; IGRP, islet-specific glucose-6-phosphatase catalytic subunit-related protein.

**Figure 6:**
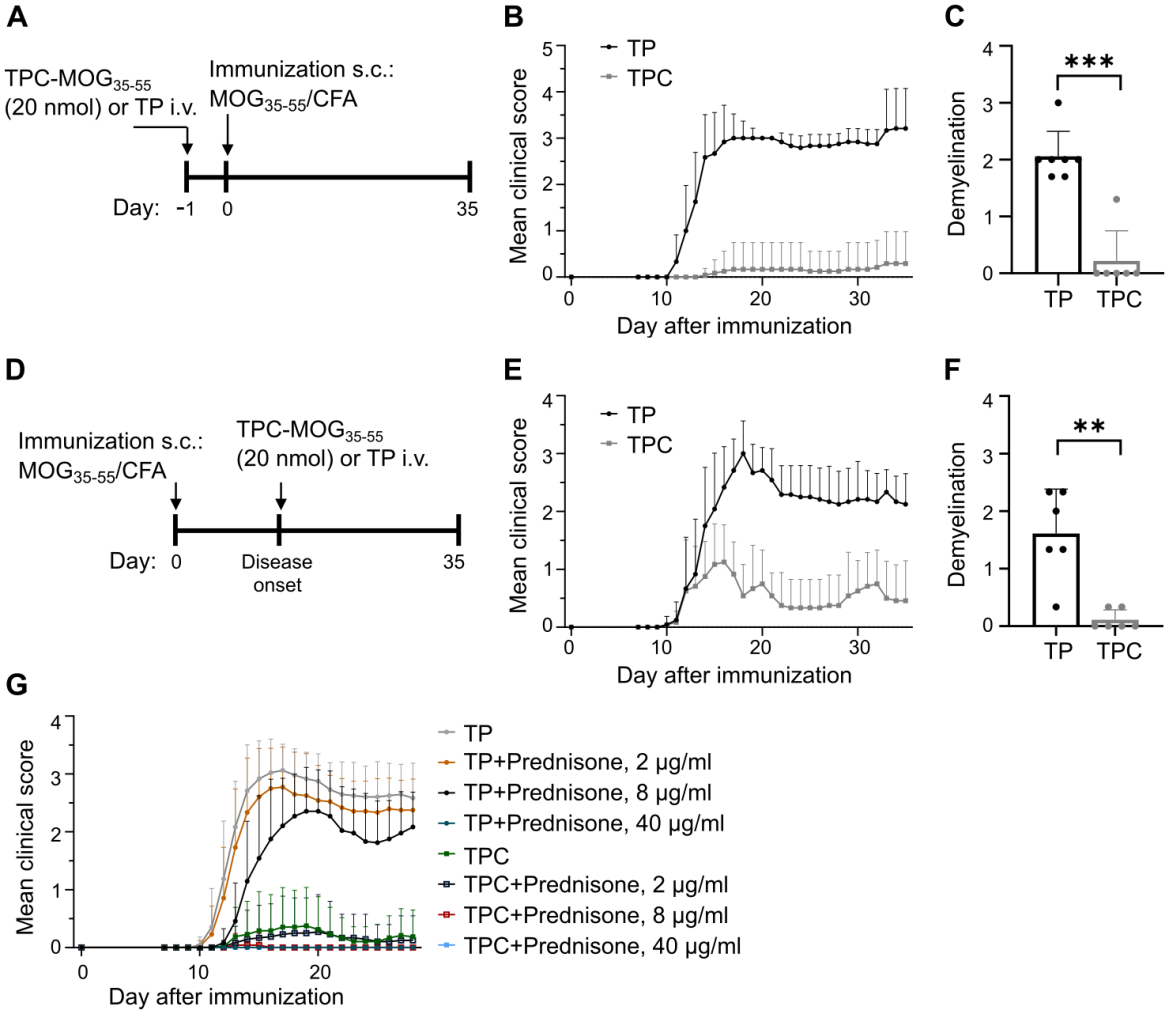
TPCs prevent or alleviate EAE with significant reduction in disease severity and demyelination in spinal cords. EAE was induced in C57B/6 mice through immunization with MOG_35-55_ peptide/CFA plus i.p. injections of pertussis toxin. TP or TPC-MOG_35–55_ (20 nmol) was administered either one day before immunization (prophylactic treatment) or at the disease onset (therapeutic treatment). The animals were observed daily until day 35. At the end of follow-up, spinal cords were collected for histological analysis. **(A–C)** Prophylactic treatment setting: study design **(A)**, mean clinical score **(B)**, and demyelination score **(C)**. **(D–F)** Therapeutic treatment setting: study design **(D)**, mean clinical score **(E)**, and demyelination score **(F)**. **(G)** Preventive treatment of EAE with TPC-MOG_35–55_ (20 nmol) with concurrent prednisone in different doses. Graphs show means ± SD of 6–12 animals per group. A representative experiment of two studies that resulted in similar outcomes is shown **(G)**. **p < 0.01; ***p < 0.001 by Mann-Whitney test for the differences in demyelination score between TP and TPC groups.

In the MOG-induced experimental autoimmune encephalomyelitis (EAE) model of MS, a single prophylactic injection of TPC-MOG_35-55_ prior to disease induction significantly attenuated disease severity, whereas TP-treated mice developed clinical symptoms (**Figures 6A, B**, **Supplementary Figures S3A–C**). Consistent with the attenuated disease scores, prophylactic treatment with TPC-MOG_35-55_ significantly mitigated demyelination within spinal cords compared to those of TP-treated mice (**Figure 6C**, **Supplementary Figures S4, A, B**). Our study also explored the therapeutic potential of TPCs. Notably, a single injection of TPC-MOG_35-55_ at disease onset significantly reduced both mean clinical scores (**Figures 6D, E**, **Supplementary Figures S3D–F**) and demyelination (**Figure 6F**, **Supplementary Figures S4, C, D**). These findings underscore the efficacy of TPCs in inducing tolerance prophylactically and therapeutically. Furthermore, this disease model was qualified as the standard *in vivo* test system for developing our platform technology due to the good reproducibility and reliability of disease induction, as well as the relative short observation period. In these EAE studies (prophylactic or therapeutic), we used either vehicle (saline or mannitol formulation buffer) or TPs as negative controls. These two negative controls exhibited comparable lack of tolerance effects (**Supplementary Figure S5**). To demonstrate that the peptide conjugated to TPs, rather than the particle themselves, is indeed the critical entity for mediating peptide-specific tolerance effects, we switched to the use of empty TPs as negative controls in subsequent studies as shown here.

### No pharmacodynamic interaction between TPCs and low-to-medium dose glucocorticoids

Glucocorticoids are widely used to treat various autoimmune conditions (19). To assess the potential impact of prednisone on the efficacy of TPCs in suppressing EAE, we administered three doses of prednisone in drinking water (2, 8, and 40 μg/ml) following prophylactic application of TP or TPC-MOG_35-55_. These doses were equivalent to the oral administration of 5, 10, and 50 mg/day in humans, respectively (20).

The data show that low-to-medium doses of prednisone (equivalent to ≤ 10 mg/day in humans for maintenance therapy) neither prevented EAE development in mice receiving TPs alone, nor impaired any protective efficacy of TPC-MOG_35-55_ (**Figure 6G**). The results were comparable to those achieved in the group receiving TPC-MOG_35-55_ without any prednisone treatment. In contrast, high-dose prednisone per se eliminated the EAE clinical phenotype, preventing any interpretation of the tolerance effects of TPCs. Altogether, the efficacy of TPCs remains unaffected by the administration of prednisone at maintenance doses.

### TPCs induce antigen-specific Tregs and T cell anergy

**Figure 7:**
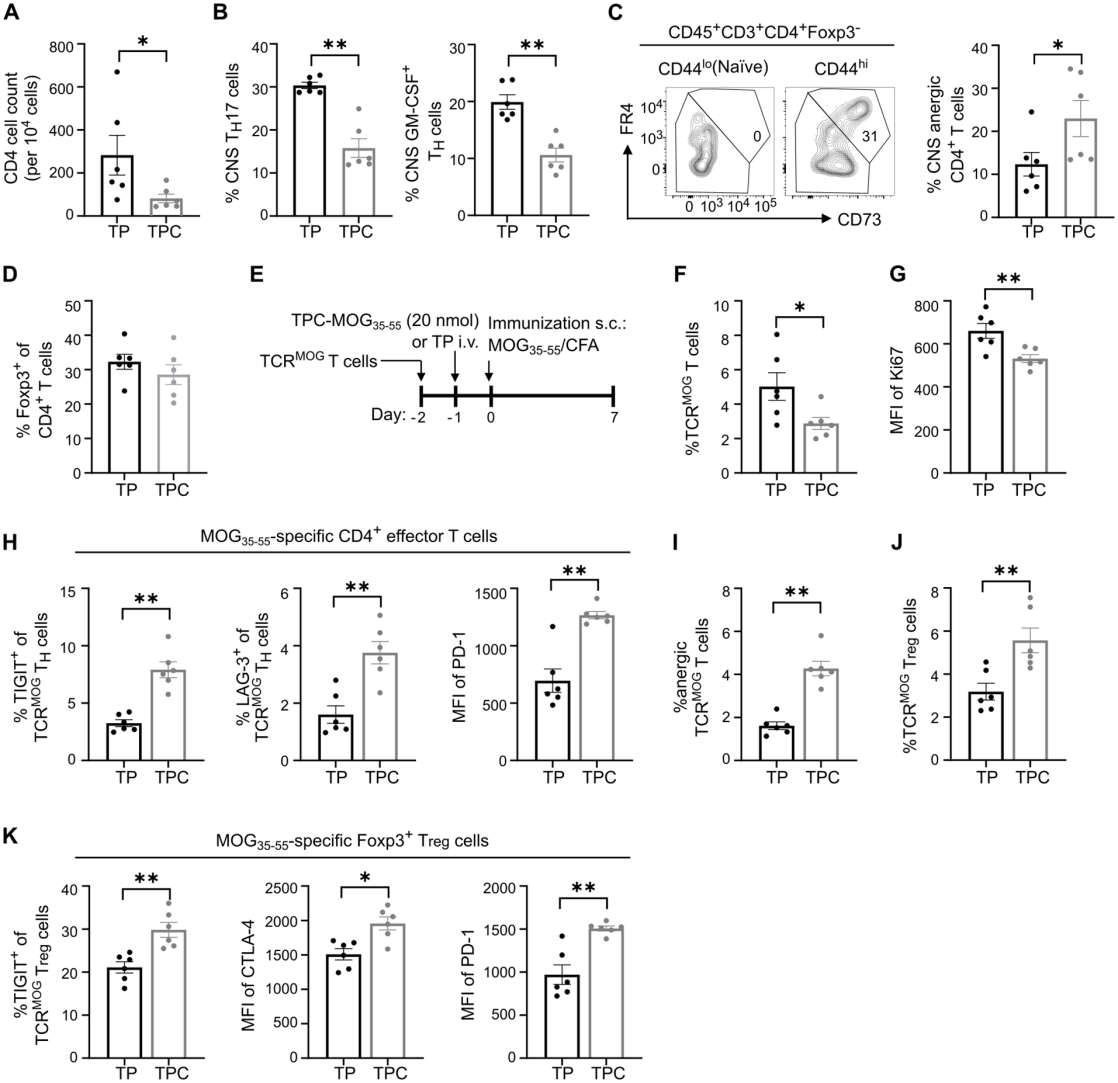
TPCs induce anergic cells and enhance expression of co-inhibitory receptors. **(A–D)** Lymphocytes were isolated from spinal cords (i.e., CNS) of the animals in EAE therapeutic setting (**Figure 6D**; gating strategy: **Supplementary Figure S2B**). Absolute CNS CD4^+^ T cell count **(A)**. Frequency of CNS T_H_17 and GM-CSF–producing T cells **(B)**. Representative flow cytometry plot of CD73^hi^FR4^hi^ subpopulation in naïve and effector T cells and frequency of anergic CD4^+^ T cells in CNS **(C)**. The gate identifying CD73^hi^FR4^hi^ cells among effector T cells (CD3^+^CD4^+^Foxp3^-^CD44^hi^) was validated against naïve T cells (CD3^+^CD4^+^Foxp3^-^CD44^lo^) to ensure no CD73^hi^FR4^hi^ cells were included in the naïve T cell population. Frequency of CNS Tregs **(D)**. **(E–K)** Adoptive transfer of TCR^MOG^ T cells into wild-type mice. Study design **(E)**. Frequency of splenic TCR^MOG^ T cells **(F)** and their median fluorescence intensity (MFI) of Ki67 **(G)**. Frequency of TIGIT^+^ and LAG-3^+^ cells among TCR^MOG^ T cells and MFI of PD-1 of TCR^MOG^ T cells in spleen **(H)**. Frequency of anergic TCR^MOG^ T cells in spleen **(I)**. Frequency of splenic TCR^MOG^ Tregs **(J)**. Frequency of TIGIT^+^ cells and MFI of CTLA-4 and PD-1 among TCR^MOG^ Tregs in spleen **(K)**. CNS, central nervous system. Graphs show means ± SD of six animals per group. Data are a summary of two independent experiments. *p < 0.05; **p < 0.01 by Mann-Whitney test.

To delve into the tolerance mechanisms, we analyzed the lymphocytes isolated from spinal cords on day 35 in the EAE study. While T_H_17 cells are considered the primary encephalitogenic T cells in EAE (21), the RORγt-driven production of GM-CSF has been identified as crucial in autoimmune neuroinflammation (22). Indeed, therapeutic administration of TPC-MOG_35-55_ mitigated CD4^+^ T cell infiltration in spinal cords by 70% and significantly reduced T_H_17 and GM-CSF-secreting T_H_ cells (**Figures 7A, B**). The decrease in these proinflammatory T cell subsets could derive from either their physical deletion or functional suppression, which may be associated extrinsically with Treg induction (23), or intrinsically with anergy or exhaustion (24). TPC-MOG_35-55_ also led to a significant increase in anergic cells on day 35, identified as CD3^+^CD4^+^Foxp3^–^CD44^hi^CD73^hi^FR4^hi^ subset, as previously characterized (25) (**Figure 7C**). However, the frequency of Tregs remained similar between TP and TPC groups at this late timepoint (**Figure 7D**).

To investigate the early tolerance mechanisms specifically on peptide-specific CD4^+^ T cells, we employed an adoptive transfer of TCR^MOG^ T cells (**Figure 7E**). Two days after treatment with TPC-MOG_35-55_, there were significantly more TCR^MOG^ Tregs in the liver of TPC-treated compared to TP-treated mice (**Figure 3F**). Eight days after treatment, we observed a significant reduction in the frequency of splenic TCR^MOG^ T cells and an approximate 20% reduction in their Ki67 expression, suggestive of restricted proliferation associated with treatment (**Figures 7F, G**). Moreover, upon TPC-treatment, TCR^MOG^ T cells displayed a phenotype of anergy and suppressed effector functions associated with a significant upregulation of TIGIT, LAG-3 and PD-1 compared to those of TP-treated mice (**Figures 7H, I**). Notably, we also detected a significant increase in TCR^MOG^ Tregs in TPC-treated compared to TP-treated mice (**Figure 7J**). The expression of co-inhibitory receptors on TCR^MOG^ Tregs significantly increased in TPC-treated animals, suggesting a heightened suppressive capacity of Tregs (**Figure 7K**).

## Discussion

We established the only clinical-stage nanoparticle-based platform specifically designed for leveraging the tolerogenic potentials of LSECs. Preferential LSEC targeting by TPCs resulted in effective tolerance induction across various CD4^+^ T cell-mediated disease models. This is achieved by conferring anergy to CD4^+^ T cells and inducing antigen-specific Tregs, which counteract proinflammatory T cell subsets. These findings, together with the platform’s flexibility in coupling with a wide array of disease-relevant peptides via the optimized linker technology, created the rationale for moving into clinical testing.

TPCs were specifically designed for preferential uptake by LSECs, with negligible uptake by other NPLCs. This differentiates them from other tolerizing particles, exhibiting a different organ distribution pattern (26, 27) or different cellular target specificities within the liver without predominant uptake by LSECs (9, 28, 29). We believe that our approach offers significant advantages thanks to the unique features of LSECs. In fact, their predominant location in sinusoidal blood, along with their extraordinary clearance function, facilitates rapid uptake of nanosized carriers (~100 nm) for antigen-specific immunotherapy (30). Moreover, LSECs can strongly induce Tregs (31), veto dendritic cell-induced T cell activation, and sequestrate activated CD8^+^ T cells, all of which contribute to their extraordinary tolerogenic capacity (32), outmatching other tolerogenic NPLCs. Notably, other tolerogenic NPLCs (e.g., Kupffer cells) exhibit high plasticity, inducing tolerance only under homeostatic conditions (33) but activating immune responses during inflammation (34, 35). This may limit their role as mediators of tolerance in treating ADs, where inflammatory dysfunction affects disease pathogenesis and progression (36). Conversely, LSECs have demonstrated remarkable resilience to external inflammatory stimuli, maintaining robustness in tolerance induction (37).

Although some TPC uptake and potential tolerance induction by other APCs cannot be excluded, the data presented in this study indicate preferential targeting of TPCs to LSECs. Previously collected pharmacokinetic data on different TPCs showed rapid clearance of TPCs from the circulation, with plasma half-lives ranging from 15 to 30 min depending on dose (14). Based on this, it can be assumed that one hour post-injection, no relevant amount of TPCs remains in the circulation, making re-targeting to other organs or cells after this timepoint unlikely. The vast majority of TPCs have been clearly taken up by LSECs well within one hour, as demonstrated by intravital microscopy. We do not exclude the possibility that Kupffer cells or other APCs in the liver or spleen may contribute to tolerance induction; however, as in all these cells TPCs accumulated only in a small amount, their contribution appears less significant. To confirm this notion, we had previously administered the former version of the current nanoparticles to splenectomized mice in which induction of tolerance was not impaired (14). FRET analysis further demonstrates that considerable peptide release in LSECs has already occurred one hour post-injection, while uptake by hepatocytes, stellate cells, or other hepatic APCs remained undetectable, as fluorescence signals were confined to the sinusoidal lining. This clearly indicates that LSECs are the preferential targets of TPCs up to one hour after injection, with redistribution after this timepoint deems to be unlikely.

The size and charge of TPCs are believed to contribute to their preferential targeting. Their size (25–30 nm) facilitates efficient uptake by LSECs, aligning with the dual-cell principle of waste clearance (30). Considering the narrow fenestrae of human LSECs (~100 nm (38)), TPCs are particularly suited for LSEC targeting in humans. Importantly, uniform size distribution and the absence of aggregates confirm that TPCs remain consistently small. Additionally, the negative ζ-potential of TPCs enhances their affinity for LSECs, given that scavenger receptors on LSECs bind to diverse polyanionic molecules (30). As the negative charge of nanoparticles is commonly associated with anti-inflammatory and tolerogenic properties (39), this feature may also contribute to tolerance promotion by TPCs.

Since TP conjugation is restricted to peptides, this requires the identification and characterization of immunogenic T cell epitopes and their corresponding MHC molecules. The direct delivery of disease-relevant peptides to LSECs appears advantageous, as i) TPCs carry considerably higher doses of relevant T cell epitopes to LSECs compared to nanoparticles delivering full proteins, comprising proportionally fewer peptide-epitopes; ii) Manifold peptides can be coupled due to a versatile linker technology; iii) TPMs deliver a combination of selected peptides from multiple antigens, thereby amplifying the tolerization effect; iv) Several ADs are linked to certain MHC molecules, facilitating patient-specific peptide selection. Taken together, TPC technology has the potential for a personalized approach, targeting a well-defined patient population with a precise and disease-relevant tolerization strategy.

To evaluate potential pharmacodynamic interactions between TPCs and glucocorticoids, a mainstay of AD treatment, we investigated the effect of the concomitant administration of TPCs and glucocorticoids in mice with MOG-induced EAE. The data show that TPC’s efficacy remained unaffected by prednisone at doses equivalent to those used for human maintenance therapy (40). Therefore, the mechanisms promoting TPC-mediated tolerization appeared unaffected by the nonspecific immunosuppressive and anti-inflammatory effects of glucocorticoids, which modulate immune responses through glucocorticoid receptor-mediated gene regulation and transcription factor inhibition (41). Importantly, although high-dose prednisone effectively suppressed EAE symptoms, TPC treatment alone could achieve similar outcomes without the complications associated with glucocorticoids. Furthermore, high-dose glucocorticoids are not expected to be used as maintenance therapy in clinical practice. These findings are highly relevant for future steps in the assessment of TPCs in AD patients, who often require glucocorticoids.

Mechanistically, TPCs initially activated MOG-specific T cells but halted their further proliferation upon encountering cognate antigens. These effector cells upregulated anergic markers and various co-inhibitory receptors. The observed phenotypes and reduced proliferative capacity align with typical features of anergy and exhaustion (24, 25), suggesting a probable link between TPC-induced tolerance and these functional impairments. Anergy typically arises from T cell activation upon TCR recognition lacking adequate co-stimulation (42). As LSECs express low levels of MHC-II and co-stimulatory molecules due to the tolerogenic microenvironment of the liver (12, 43), their interaction with antigen-specific T cells, whose specificity corresponds to the TPC-delivered peptides, might promote T cell anergy.

Notably, other work shows that the anergy markers CD73 and FR4 were expressed on antigen-exhausted CD4^+^ T cells in a dose-dependent manner, with gene expression by antigen-exhausted cells highly enriched in anergic cells as well (24). Thus, anergy and exhaustion share common features despite conceptual differences in T cell priming, and essentially both are associated with quality and quantity of TCR signal strength (24, 44). This in turn can be effectively influenced through selection of peptides coupled to TPCs to induce these dysfunctional states, thereby facilitating tolerance.

In line with the role of Tregs in preventing EAE (14), our data furthermore demonstrate that TPC treatment significantly increased Tregs within the antigen-specific compartment of CD4^+^ T cells in the liver and spleen shortly after treatment. As it appears, antigen-specific Tregs were induced in the liver first and then circulated to the spleen, since their increase was observed in the liver before the spleen post-treatment. At a later timepoint, there was no significant increase in Tregs, but, strikingly, we observed a significant increase in anergic T cells and a significant reduction inT_H_17 and GM-CSF–producing T_H_ cells in the spinal cords of TPC-treated animals. These findings underscore the TPC-mediated tolerization effects in suppressing disease-relevant proinflammatory cells in the target organ. The mechanism through which tolerance, once induced, is maintained remains to be elucidated.

Besides antigen-specific Treg induction in the EAE model, we also observed an increase in insulin-specific Tregs in the spleens of animals receiving TPM-T1D before diabetes onset together with a reduction in T1D incidence. This suggests that inducing disease-relevant Tregs could slow disease progression. Consistent with these observations, we previously identified significantly reduced insulin-specific Tregs in the blood of children at the onset of islet autoimmunity (45) and linked increased insulin-specific Tregs with slow progression to symptomatic T1D (46). While clinical studies have failed to show protein- or peptide-mediated tolerance induction in adults with established T1D, the potential for antigen-specific tolerization to increase insulin-specific Tregs in the early T1D stages was highlighted in the Pre-POINT study (47). However, while insulin dominates the autoimmune reaction in NOD mouse (17), the situation is less clear in human T1D. As multiple autoantigens are likely involved in the pathogenesis, a broader tolerance induction approach administering several autoantigens simultaneously may be more beneficial (18). Our present study has evidently confirmed this by applying five different T cell epitopes coupled to TPCs (with each peptide administered in the same dose range comparable to the other preclinical studies). However, due to flow cytometry panel size limitations, further investigation into Tregs specific to antigens other than insulin is warranted to address their role in tackling pathogenicity.

Although the EAE, DTH, and T1D models used here share key immunopathological features with the respective human diseases, they primarily served to demonstrate proof of concept for tolerance induction in the development of clinical-stage TPCs. However, direct clinical translation may be limited by potential immunological and physiological differences between these models and human conditions. Thus, for future clinical development, for example, in MS or T1D, additional non-clinical studies are warranted to further evaluate the efficacy and safety of TPCs for each respective disease indication.

In conclusion, we demonstrated that the optimization of TPCs enabled GMP-compliant manufacturing and their conjugation with a variety of peptides using the proprietary linker technology, ensuring the platform’s versatility for clinical testing in various indications. Thanks to their physicochemical features, TPCs primarily target LSECs, where efficient peptide release occurs, triggering the inherent tolerogenic mechanisms of these unconventional APCs. Their efficacy in inducing antigen-specific immune tolerance has been validated in various animal models. We believe that our approach represents an innovative treatment option for reinstating antigen-specific tolerance in ADs, while avoiding the complications of broad immunosuppression. TPCs are in clinical development for pemphigus vulgaris (EudraCT:2019-001727-12) and coeliac disease (NCT05660109).

## Materials and methods

### Preparation of antigen peptide-loaded nanoparticles

The preparation of nanoparticles involved encapsulating oleic acid-stabilized SPIONs into an amphiphilic polymer, namely poly(maleic acid-*alt*-1-octadecene), as previously described (48). Polymer-coated SPIONs were coupled with peptides in the presence of EDC (1-ethyl-3-(3-dimethylaminopropyl)-carbodiimide). A 150-fold excess of the relevant peptide was added, followed by incubation for 2.5 h at room temperature. The free peptide was removed using centrifugal ultrafiltration (molecular weight cut-off 100 kDa, 4200 rpm, 25°C). The hydrodynamic size of a nanoparticle was determined by dynamic light scattering using Zetasizer Nano ZS (Malvern Panalytical) and confirmed after each step of the process: before coupling, after coupling, after purification, and after formulation. Furthermore, the size distribution of the starting material (i.e., TP) was verified by Transmission electron microscopy. Peptide content and coupling efficiency were determined using AccQ Tag derivatization method for amino acid analysis (Waters Corporation). The individual TPCs were dispersed, mixed, and diluted in D-Mannitol (5% w/v), L-Lactic Acid (6 mM), and TRIS (5 mM), also referred to as MTL buffer to obtain the respective doses.

### Animal studies

Animal studies including intravital microscopy, organ distribution, and targeted delivery of TPCs, EAE, DTH, and type 1 diabetes were approved by the respective review boards of University Medical Center Hamburg-Eppendorf, Hooke Laboratories, Evotec SE, and Helmholtz Munich. All mice were bred and housed in specific pathogen-free conditions. The execution of animal procedures adhered strictly to both local and national guidelines and regulations.

### Intravital microscopy

Intravital microscopy was performed as previously described (49). Briefly, C57BL/6 mice were anesthetized using 2% isoflurane (inhalation at 2 L/min) via a small animal anesthetic face mask, and a tail vein was catheterized. The liver was exposed and mounted on a cover glass. Microscopy was performed using a Nikon A1 confocal microscope with a resonant scanner for image acquisition at 30 fps. For visualization of TPC targeting, Cy5-labelled nanoparticles were injected via tail vein catheter and the uptake into the liver was monitored for 15 min.

### Organ and liver-specific cellular distribution of TPCs

C57BL/6 mice were intravenously injected with TPCs coupled with Cy5-labelled gliadin peptides and sacrificed after 10 min. For organ distribution analysis, following whole cardiac perfusion with phosphate-buffered saline, the liver, spleen, kidneys, lung, spinal cords, and inguinal lymph nodes were harvested, homogenized, and fluorescence was measured using a Tecan microplate reader (excitation: 633 nm, emission: 670 nm). For liver-targeting analysis, NPLCs were isolated by density gradient centrifugation as described previously (31). Briefly, mouse livers were perfused with 1 mg/ml collagenase type 2 (Worthington) in Gey’s balanced salt solution, mechanically dissected, and then digested for 25 min at 37°C in 1 mg/ml collagenase type 2 solution. NPLCs were recovered using a 17% Optiprep (Sigma-Aldrich) gradient after removing hepatocytes by centrifugation. Cells were stained and analyzed by flow cytometry.

### DTH in response to Ova_323-339_

A 24-h ear swelling assay was performed to assess DTH reactions in female BALB/c mice (Janvier Labs), which were immunized s.c. with 100 μl of Ova_323-339_ peptides in CFA (1 mg/ml) seven days before the assay (50). Baseline ear thickness was measured using calipers for both ears. Immediately afterward, each mouse received an intradermal injection of Ova_323-339_ peptides (10 μg in 10 μl of PBS), or 10 μl of PBS alone as a vehicle control, into the bilateral ears to elicit DTH responses or serve as a control, respectively. The increase in ear thickness from baseline was determined 24 hours after the peptide challenge, denoted as ear swelling (μm). Δ Ear swelling was calculated by subtracting the ear swelling of vehicle control from that of the peptide-challenged ear in each animal. Following this, each ear pinna underwent a puncture biopsy, and the 8-mm–diameter biopsied tissue was weighed. The difference between the weight of the peptide-challenged ear and the control was determined, denoted as Δ ear weight (mg). The experiments were conducted at Evotec SE.

### Chronic EAE model

C57BL/6 mice (Taconic Biosciences) were immunized with MOG_35-55_ peptide in CFA emulsion, followed by i.p. injection of pertussis toxin as described previously (51). Depending on the treatment arms, the animals received either TP or TPC-MOG_35-55_ one day before immunization (prophylactic treatment) or at the onset of disease (therapeutic treatment). For the studies assessing the effects of TP versus TPC-MOG_35-55_ in the presence of glucocorticoids, prednisone was administered *ad libitum* in drinking water from Day -5 (five days before immunization) through Day 28 (the end of the study). Prednisone stock solution was prepared fresh daily at 3 mg/mL in 100% ethanol and was then diluted in drinking water to reach the final concentration of 2 mg/mL, 8 mg/mL, and 40 mg/mL, respectively. For the duration of treatment, all drinking water was replaced at the same time (+/- 1 hour) each day (52). Individual animals were observed daily until day 35, and blinded EAE scoring was conducted on a scale of 0 to 5: 0 = asymptomatic; 1 = limp tail; 2 = hind limb weakness; 3 = hind limb paralysis; 4 = hind limb paralysis plus partial front limb paralysis; 5 = 4-limb paralysis or death. The data are presented as the mean clinical score. At the end of the follow-up, the spinal cord and hindbrain were collected for flow cytometry analysis or histological analysis. The experiments were conducted at Hooke Laboratories.

### *In vitro* stimulation of MOG-specific T cells

Spleen and lymph node cells (inguinal, axillary, and brachial) were isolated from TCR^MOG^ CD45.2 mice (C57BL/6-Tg(Tcra2D2,Tcrb2D2)1Kuch/J), followed by erythrocyte depletion with ACK lysis buffer (Thermo Fisher Scientific). 5x10^5^ isolated cells were cultured in 200 µl of full RPMI 1640 (PAN-Biotech) supplemented with 10% heat-inactivated fetal bovine serum (Corning) at 37°C for 72 h in the presence of either MOG_35-55_ peptide or TPC-MOG_35-55_. The supernatant was collected for quantification of IFNγ using ELISA kit (R&D Systems).

### Adoptive transfer of 2D2 TCR transgenic cells

Spleen and lymph node cells (inguinal, axillary, and brachial) were isolated from TCR^MOG^ CD45.2 mice (C57BL/6-Tg(Tcra2D2,Tcrb2D2)1Kuch/J), followed by enrichment of CD4^+^ T cells through negative immunomagnetic selection (Dynabeads™ Untouched™ Mouse CD4 Cells Kit, Thermo Fisher Scientific). Subsequently, 8x10^6^ CD4^+^ T cells were i.v. injected into C57BL/6 mice with congenic marker CD45.1. In some experiments, the cells were labelled with CFSE (BioLegend) before adoptive transfer. The day after the adoptive transfer, recipient mice were treated with either TP or TPC-MOG_35-55_. One day post-treatment, recipient mice were immunized s.c. with 100 mg of MOG_35-55_ peptide in CFA. Seven days after immunization, spleens and lymph nodes were harvested for flow cytometry analysis. TCR^MOG^ T cells within these tissues were identified using CD45.2 and examined for a range of surface and intracellular markers.

### The NOD mouse model of type 1 diabetes

Female NOD/ShiLtJ mice were purchased from The Jackson Laboratory at 4 weeks of age and were acclimated to the facility at Helmholtz Munich before the start of the experiments. At the age of 8 weeks, the animals received either TP or TPM-T1D: a mix of five TPCs, conjugated respectively with 50 nmol of diabetogenic peptides (Insulin beta chain 9–23, Proinsulin p24–33, islet-specific glucose—phosphate catalytic subunit-related protein 206–214, 2.5 hybrid insulin peptide and 6.9 hybrid insulin peptide) (**Table 2**). The animals received five weekly injections of TPs or TPM-T1D followed by two bi-weekly injections for a total of seven nanoparticle injections. Blood sugar levels were determined weekly until the end of the experiment when mice reached an age of 24 weeks. Mice with two consecutive blood sugar readings ≥ 250 mg/dL were considered diabetic and euthanized by CO_2_ in accordance with animal protocols approved by the government of upper Bavaria. Whenever possible, the mouse was euthanized directly in its home cage, with nesting material and shelter removed in advance. Euthanasia by CO_2_ was carried out using a Quietek system, which ensured gradual filling at a flow rate of 20% of the cage volume per minute, with a final concentration of 55% CO_2_ of chamber volume per minute. A baffle plate is fitted in the cage lid to ensure even distribution of CO_2_. The mouse remained in the cage for at least five minutes. Following removal from the CO_2_, death was ensured by perfusion. Spleens of all mice were harvested and analyzed via flow cytometry following euthanasia either due to hyperglycemia or at the end of the experiment.

### Flow cytometry analysis

Single-cell suspension (containing immune cells) was prepared from different organs such as liver, spleen, and spinal cords, depending on the experimental design. For NOD mouse model, unspecific binding of antibodies was prevented by incubation of single-cell suspensions with Fc-Block (BD Pharmingen, 2.4G2, 1:100) in RPMI with 10% heat-inactivated FCS for 10 min on ice, followed by tetramer staining with 15 µg/ml PE-labelled insulin-specific or control tetramers for 1h at 37°C. Tetramer complexes were composed of MHC-II Iag7 and mimotope peptides derived from Insulin-B:9-23 (sequences: HLVERLYLVCGGEG and HLVERLYLVCGEEG; NIH Tetramer Core Facility) (46). Antibodies for surface staining (**Supplementary Table S1**) were added immediately after tetramer staining without washing and incubated for 30 min on ice in the dark. For EAE and 2D2 transfer models, tetramer staining was skipped. Before staining for cytokines, cells were cultured with phorbol myristate acetate (50 ng/ml), ionomycin (1 µg/ml), monensin (1.36 µg/ml), and brefeldin-A (1 µg/ml) for 4 h; all reagents were purchased from Sigma-Aldrich. To detect cytokines and other intracellular proteins such as Foxp3 and Ki-67 (**Supplementary Table S1**), T cells were fixed and permeabilized using Foxp3/Transcription Factor Staining Buffer Set (eBioscience™, Thermo Fisher Scientific) after surface staining. Intracellular proteins were stained for 30 min on ice in the dark and washed 2-3 times with Perm buffer and 1-2 times with HBSS+ (Hanks balanced salt soluation supplemented with 5% heat-inactivated FCS) before acquisition on the flow cytometer. Cells were acquired on a BD FACSAriaIII or a LSRFortessa™ flow cytometer (depending on the facility where the experiments were conducted) using FACSDiva software with optimal compensation and gain settings determined for each experiment based on unstained and single-color stained samples. Fluorescence minus one (FMO) controls were used to set gates. Doublets were excluded based on SSC-A vs. SSC-W plots and FSC-A vs. FSC-W plots. Live cell populations were gated based on cell side and forward scatter and the exclusion of cells positive for Fixable Viability Dye eFluor450 (eBioscience). Samples were analyzed using FlowJo software version 10.8.1 (TreeStar Inc.).

### RNA extraction from ear pinnae and real-time reverse transcription-quantitative PCR

The ear pinnae were stored at –80°C in RNAlater solution (Thermo Fisher Scientific). The thawed tissues were homogenized using a rotor-stater homogenizer, and RNA was isolated using Rneasy Fibrous Tissue Mini Kit (Qiagen) per manufacturer’s protocol. Real-time RT-qPCR was employed to compare gene expression levels in ear pinnae. The cDNA was reverse transcribed from the extracted RNA and underwent a qPCR reaction using the QuantStudio™ qPCR System (Thermo Fisher Scientific) following the manufacturer’s protocol. The primers used were all purchased from Thermo Fisher Scientific: *Gapdh* (Mm99999915_g1), *Ifng* (Mm01168134_m1), *Tnf* (Mm00443258_m1), *Il2* (Mm00434256_m1), *Il1b* (Mm00434228_m1), *Csf2* (Mm01290062_m1), *Il6* (Mm00446190_m1), *Ccl2* (Mm00441242_m1), *Ccl5* (Mm01302427_m1).

### Histological analysis for encephalomyelitis and demyelination in EAE

Spines were dissected from mice and fixed in 10% formalin. Sections with samples from cervical, thoracic, and lumbar regions of spinal cords were prepared and underwent H&E and anti-MBP staining, respectively. H&E-stained slides were used to count inflammatory foci in spinal cords, while anti-MBP-stained slides were used to estimate the demyelinated area.

### Statistical analysis

GraphPad Prism (Version 10) software was employed to perform the statistical analyses. Sample sizes were determined based on prior experience from similar experiments, without using statistical methods for pre-determination. Whenever feasible, mice were age-matched and randomly allocated to experimental groups. Experiments were conducted without knowledge of treatment to ensure blinding. *In vivo* experiments included biological replicates, with the number of replicates (n values) specified in the figure legends. The nonparametric Mann-Whitney test was used to compare outcomes between two independent groups. For survival data (normoglycemic “survivor”), a log-rank test was conducted. Comparisons were considered significant if *p <*0.05.

## Data Availability

The original contributions presented in the study are included in the article/**Supplementary Material**. Further inquiries can be directed to the corresponding authors.

## Supplementary material

Supplementary Figure 1 FRET effect exists only between QD and coupled Cy5-labelled Ova peptides. Please insert here the following text: (A) Schematic explanation of FRET. (B–C) The liver of C57BL/6 mice was examined under intravital microscopy 1 h after intravenous administration of various nanoparticles, specified respectively in the figure. **(B)** No FRET effect between TPC (iron core) and coupled Cy5-labelled Ova peptides. (*Upper* panel) 488 nm excitation does not induce QD emission, or via FRET, Cy5 emission. (*Lower* panel) 638 nm excitation leads to Cy5 emission. **(C)** No FRET effect between QD and uncoupled Cy5-labelled Ova peptides. (*Upper* panel) 488 nm excitation induces QD emission, but no Cy5 emission. (*Lower* panel) 488 nm and 638 nm excitation induce QD and Cy5 emission, respectively.

Supplementary Figure 2 Flow cytometry gating strategy. **(A)** For liver non-parenchymal cells. DC, dendritic cells; KC, Kupffer cells; LSEC, liver sinusoidal endothelial cells; MoMF, monocyte-derived macrophages; PMN, polymorphonuclear neutrophils. **(B)** For cytokine producing T cells.

Supplementary Figure 3 TPCs significantly attenuate disease scores in both prophylactic and therapeutic treatment. **(A–C)** Prophylactic treatment. End score **(A)**. Maximum score **(B)**. Cumulative score **(C)**. **(D–F)** Therapeutic treatment. End score **(D)**. Maximum score **(E)**. Cumulative score **(F)**. Graphs show means ± SD of 12 animals per group. Data are a summary of two independent experiments. *****p <*0.0001 by Mann-Whitney test for the differences in disease score between TP and TPC groups.

Supplementary Figure 4 TPCs alleviate demyelination in spinal cords of TPC-treated animals. Immunohistochemical staining of spinal cords using anti-MBP (myelin basic protein) antibody. Demyelination is observed as clearly visible unstained areas in white matter tracts and is associated with the presence of large vacuoles. **(A, B)** Prophylactic treatment setting. One representative image of the spinal cord slice from mice treated with TP **(A)** and with TPC-MOG_35–55_ (**B**). (**C**–**D**) Therapeutic treatment setting. One representative image of the spinal cord slice from mice treated with TP **(C)** and with TPC-MOG_35–55_ **(D)**. N = 6 per group. A representative experiment of two studies that resulted in similar outcomes is shown.

Supplementary Figure 5 Comparison of negative controls in EAE using either vehicle or TP. EAE was induced in C57B/6 mice through immunization with MOG_35-55_ peptide/CFA plus i.p. injections of pertussis toxin. TPC-MOG_35–55_ (14 nmol) or vehicle (saline) or TP was administered one day before immunization (prophylactic treatment). The animals were observed and scored daily until day 37. Graphs show means ± SD of eight animals per group.
