# Supplementary figures and images for: Nanoparticle platform preferentially targeting liver sinusoidal endothelial cells induces tolerance in CD4+ T cell-mediated disease models
Source: Front Immunol. 2025 Mar 17;16:1542380. doi: 10.3389/fimmu.2025.1542380 (PMC11955608; doi:10.3389/fimmu.2025.1542380)

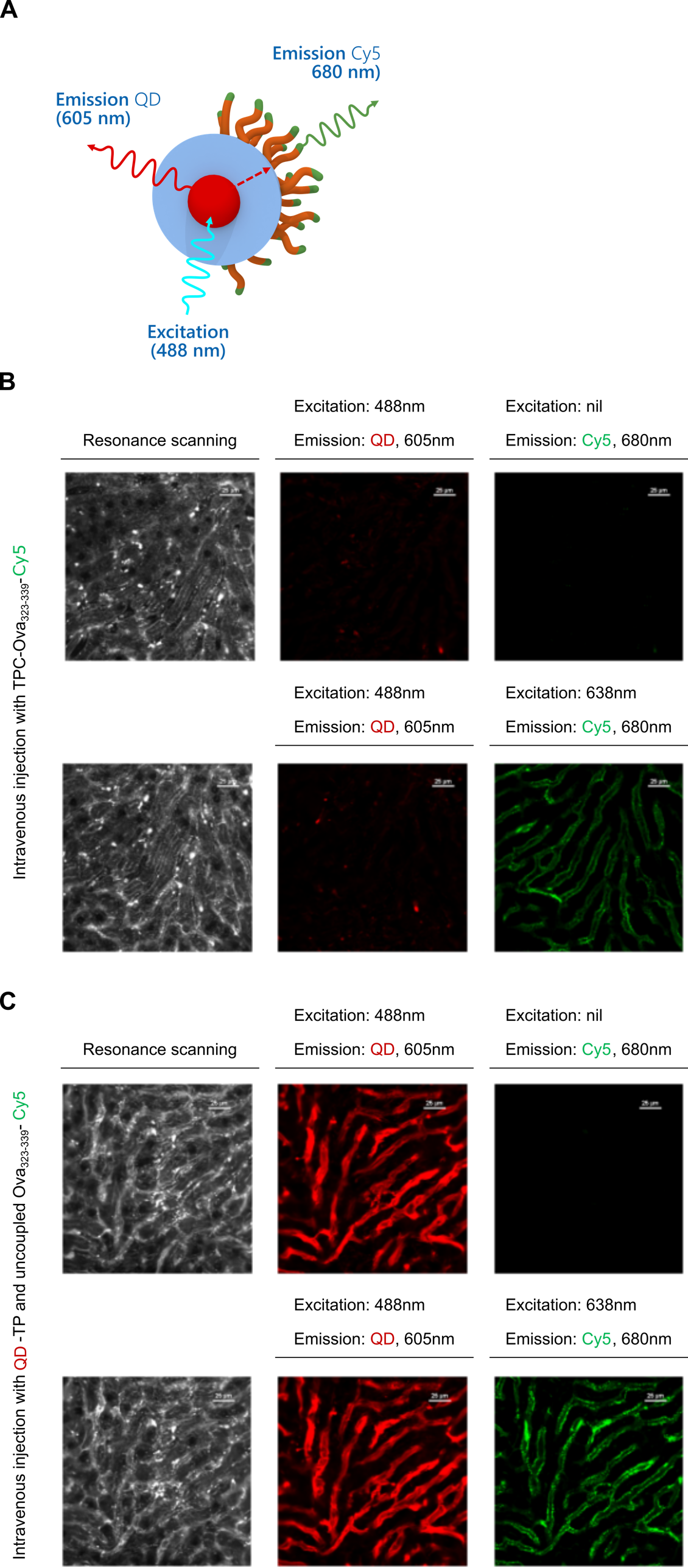

Supplement: Supplementary Figure 1 — FRET effect exists only between QD and coupled Cy5-labelled Ova peptides. Please insert here the following text: (A) Schematic explanation of FRET. (B–C) The liver of C57BL/6 mice was examined under intravital microscopy 1 h after intravenous administration of various nanoparticles, specified respectively in the figure. (B) No FRET effect between TPC (iron core) and coupled Cy5-labelled Ova peptides. (Upper panel) 488 nm excitation does not induce QD emission, or via FRET, Cy5 emission. (Lower panel) 638 nm excitation leads to Cy5 emission. (C) No FRET effect between QD and uncoupled Cy5-labelled Ova peptides. (Upper panel) 488 nm excitation induces QD emission, but no Cy5 emission. (Lower panel) 488 nm and 638 nm excitation induce QD and Cy5 emission, respectively. [file Image1.png]

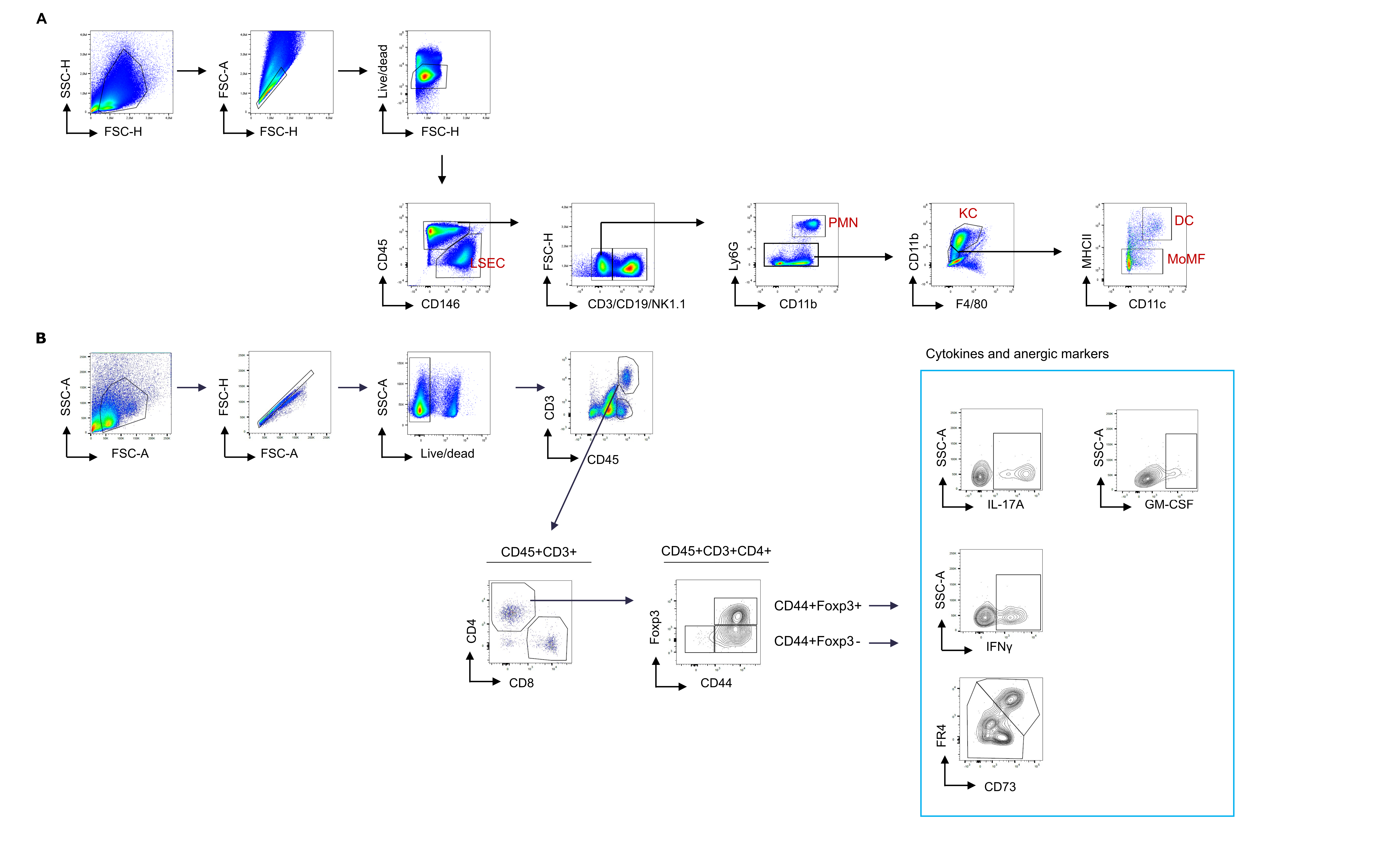

Supplement: Supplementary Figure 2 — Flow cytometry gating strategy. (A) For liver non-parenchymal cells. DC, dendritic cells; KC, Kupffer cells; LSEC, liver sinusoidal endothelial cells; MoMF, monocyte-derived macrophages; PMN, polymorphonuclear neutrophils. (B) For cytokine producing T cells. [file Image2.png]

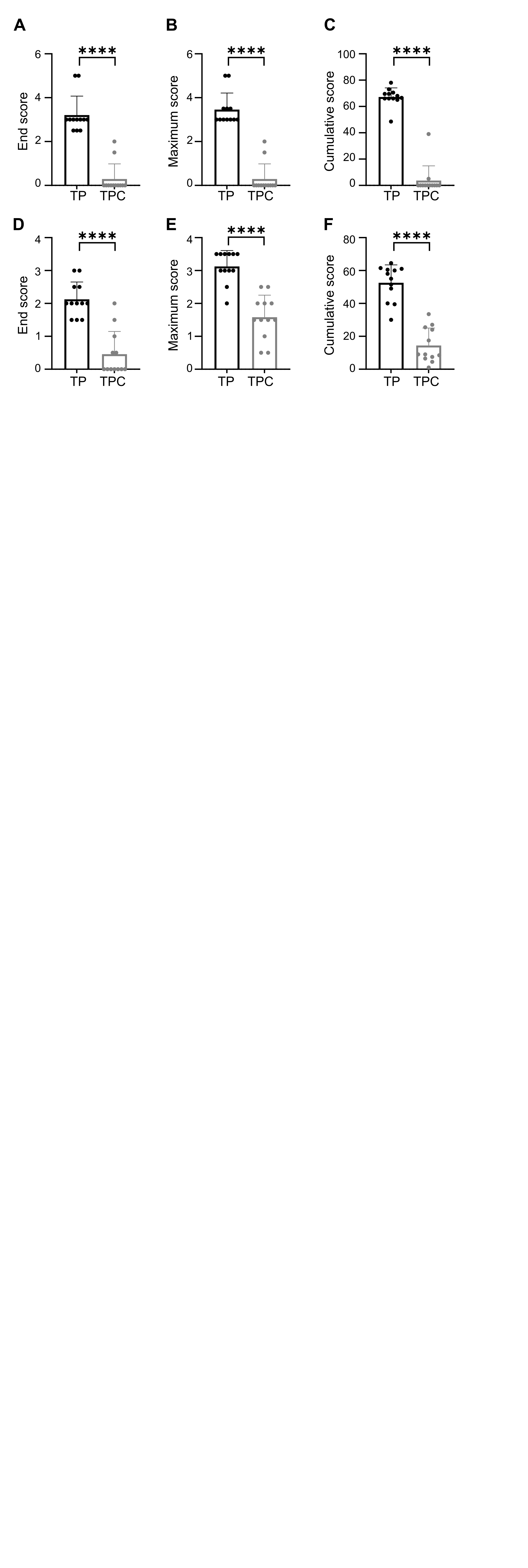

Supplement: Supplementary Figure 3 — TPCs significantly attenuate disease scores in both prophylactic and therapeutic treatment. (A–C) Prophylactic treatment. End score (A). Maximum score (B). Cumulative score (C). (D–F) Therapeutic treatment. End score (D). Maximum score (E). Cumulative score (F). Graphs show means ± SD of 12 animals per group. Data are a summary of two independent experiments. ****p <0.0001 by Mann-Whitney test for the differences in disease score between TP and TPC groups. [file Image3.png]

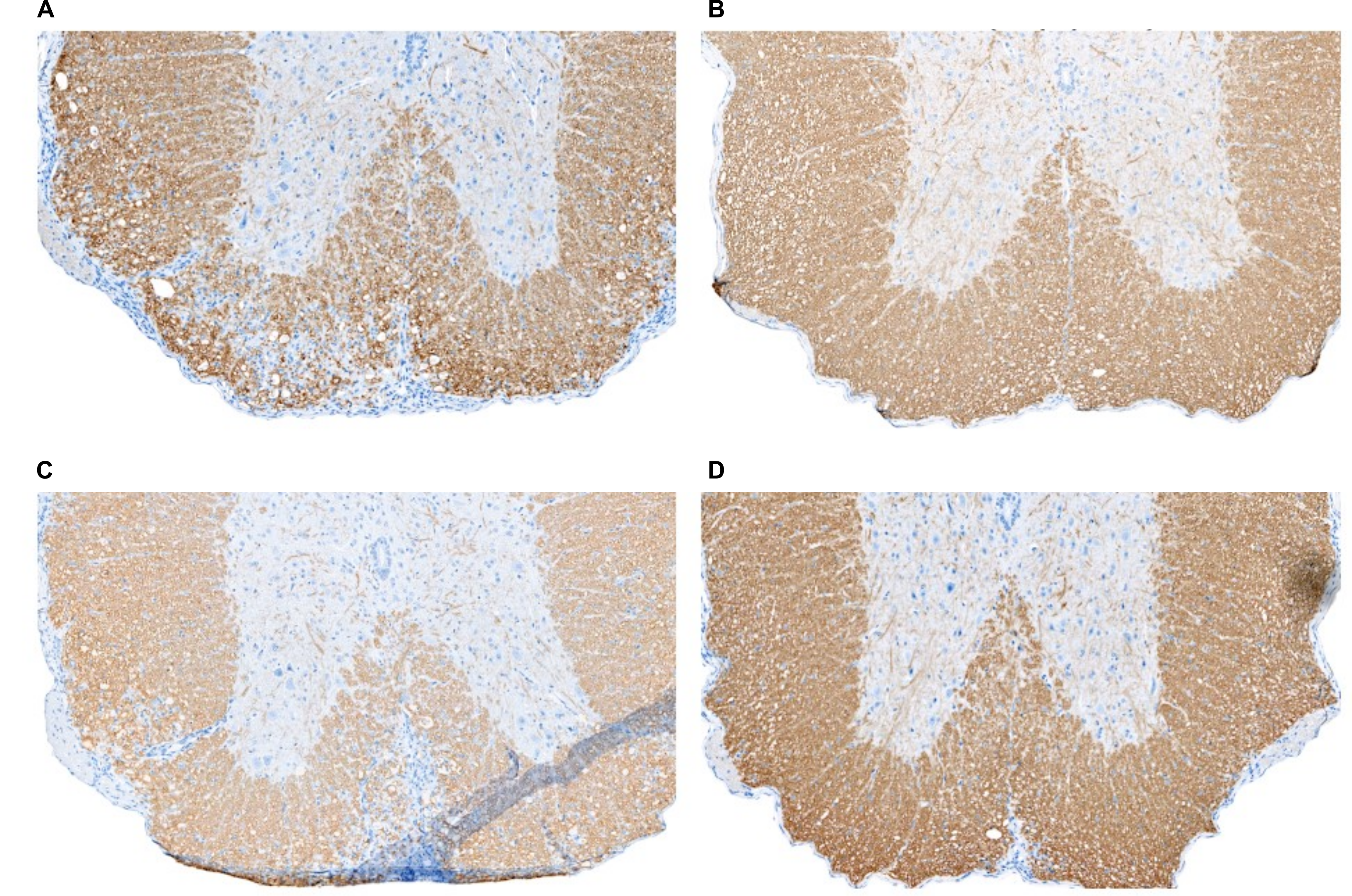

Supplement: Supplementary Figure 4 — TPCs alleviate demyelination in spinal cords of TPC-treated animals. Immunohistochemical staining of spinal cords using anti-MBP (myelin basic protein) antibody. Demyelination is observed as clearly visible unstained areas in white matter tracts and is associated with the presence of large vacuoles. (A, B) Prophylactic treatment setting. One representative image of the spinal cord slice from mice treated with TP (A) and with TPC-MOG35–55 (B). (C–D) Therapeutic treatment setting. One representative image of the spinal cord slice from mice treated with TP (C) and with TPC-MOG35–55 (D). N = 6 per group. A representative experiment of two studies that resulted in similar outcomes is shown. [file Image4.png]

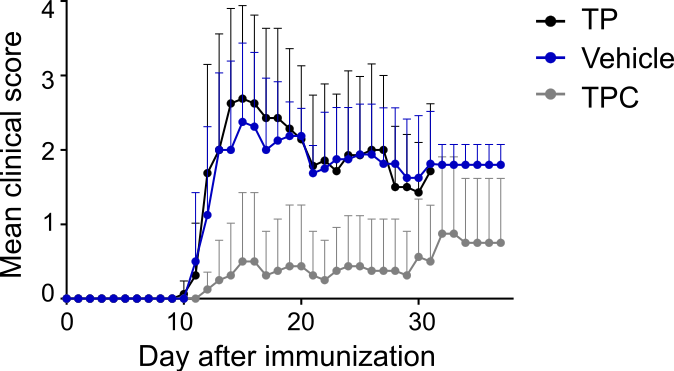

Supplement: Supplementary Figure 5 — Comparison of negative controls in EAE using either vehicle or TP. EAE was induced in C57B/6 mice through immunization with MOG35-55 peptide/CFA plus i.p. injections of pertussis toxin. TPC-MOG35–55 (14 nmol) or vehicle (saline) or TP was administered one day before immunization (prophylactic treatment). The animals were observed and scored daily until day 37. Graphs show means ± SD of eight animals per group. [file Image5.tiff]
