# Supplementary material for: Nanoparticle platform preferentially targeting liver sinusoidal endothelial cells induces tolerance in CD4+ T cell-mediated disease models
Source: Front Immunol. 2025 Mar 17;16:1542380. doi: 10.3389/fimmu.2025.1542380 (PMC11955608; doi:10.3389/fimmu.2025.1542380)
Supplement: Supplementary file 6 [file Table1.docx]

## Supplementary Tables

| **Table S1. Anti-mouse conjugated monoclonal antibody** | | |  |
| --- | --- | --- | --- |
| Specificity | Fluorochrome | Clone | Manufacturer |
| EAE | | | |
| CD45 | BV510 | 30-F11 | Biolegend |
| CD3 | PE-Cy5 | 17A2 | Biolegend |
| CD4 | Pacific blue | RM4-5 | Biolegend |
| CD44 | FITC | IM7 | Biolegend |
| CD73 | BV711 | TY/11.8 | BD biosciences |
| FR4 | PE-Cy7 | 12A5 | Biolegend |
| Foxp3 (intracellular) | APC | FJK-16s | Thermo Fisher |
| IL-17A (intracellular) | PE-Dazzle594 | TC11-18H10 | Biolegend |
| GM-CSF (intracellular) | PE | MP1-22E9 | Biolegend |
| 2D2 adoptive transfer | | | |
| CD45.2 | BV421, PE | 104 | Biolegend |
| CD3 | Pacific blue | 17A2 | Biolegend |
| CD4 | AF532 | RM4-5 | Thermo Fisher |
| CD44 | PerCP-Cy5.5 | IM7 | Biolegend |
| Foxp3 (intracellular) | APC | FJK-16s | Thermo Fisher |
| Ki67 (intracellular) | FITC | B56 | BD biosciences |
| PD-1 | PE-Dazzle594 | 29F.1A12 | Biolegend |
| CTLA-4 (intracellular) | PE | UC10-4B9 | Biolegend |
| CD73 | PE-Cy7 | TY/11.8 | Biolegend |
| FR4 | FITC | 12A5 | Biolegend |
| TIGIT | BV421 | 1G9 | Biolegend |
| LAG-3 | PE-Dazzle594 | C9B7W | Biolegend |
| Specific cellular targeting in liver | | | |
| CD11b | BUV395 | M1/70 | BD biosciences |
| CD11c | BUV737 | HL3 | BD biosciences |
| CD45 | BV711 | 30-F11 | Biolegend |
| CD146 | PE-Cy7 | ME-9F1 | Biolegend |
| F4/80 | FITC | BM8 | Biolegend |
| MHC-II | BV421 | AF6-120.1 | BD biosciences |
| Ly6G | PE | 1A8 | Biolegend |
| CD3 | PE-Cy5 | 17A2 | Biolegend |
| CD19 | PE-Cy5 | 6D5 | Biolegend |
| NK1.1 | PE-Cy5 | PK136 | Biolegend |
| NOD mouse model for T1D | | | |
| Tetramer InsB9-23 | PE | - | NIH Tetramer Core Facility |
| CD3 | BV711 | 145-2C11 | BioLegend |
| CD4 | Alexa Flour 700 | RM4-5 | BioLegend |
| Foxp3 (intracellular) | FITC | FJK-16s | Thermo Fisher |
| CD62L | BV510 | MEL-14 | BioLegend |
| PD-1 | APC-Cy7 | 29F.1A12 | BioLegend |
| CTLA-4 (intracellular) | BV605 | UC10-4B9 | BioLegend |
| TIGIT | PE-Cy7 | 1G9 | BioLegend |
| LAG-3 | APC | C9B7W | BioLegend |
| CD11c | BV421 | N418 | BioLegend |
| CD11b | Pacific Blue | M1/70 | BioLegend |
| B220 | Pacific Blue | RA3-6B2 | BioLegend |
| F4/80 | Pacific Blue | BM8 | BioLegend |
| CD14 | V450 | rmC5-3 | BD Biosciences |
| CD8a | Pacific Blue | 53-6.7 | BioLegend |
